# Draft Genome Sequence of Halotolerant Bacterium Chromohalobacter salexigens ANJ207, Isolated from Salt Crystal Deposits in Pipelines

**DOI:** 10.1128/MRA.00049-19

**Published:** 2019-04-11

**Authors:** Alok Kumar Srivastava, Anjney Sharma, Ruchi Srivastava, Praveen Kumar Tiwari, Alok Kumar Singh, Jagriti Yadav, Hena Jamali, Akhilendra Pratap Bharati, Anchal Kumar Srivastava, Prem Lal Kashyap, Hillol Chakdar, Murugan Kumar, Anil Kumar Saxena

**Affiliations:** a Indian Council of Agricultural Research (ICAR)–National Bureau of Agriculturally Important Microorganisms (NBAIM), Mau, Uttar Pradesh, India; b Indian Council of Agricultural Research (ICAR)–Indian Institute of Wheat and Barley Research (IIWBR), Karnal, India; University of Maryland School of Medicine

## Abstract

Chromohalobacter salexigens ANJ207 was isolated from a salt crystal and is known to tolerate up to 30% NaCl concentration. Here, we report the *de novo* draft assembly of C. salexigens ANJ207. The genome was determined to have 3.66 Mb represented in 13 scaffolds, with a total of 3,406 genes predicted.

## Abstract (precis)

Chromohalobacter salexigens ANJ207 was isolated from a salt crystal and is known to tolerate up to 30% NaCl concentration. Here, we report the *de novo* draft assembly of C. salexigens ANJ207.

## ANNOUNCEMENT

Salinity is one of the major threats affecting crop production all over the globe. It has been estimated that about 20% (45 million hectares) of irrigated land, producing one-third of the world’s food grains, is prone to salt stress (1). Salinity is reported as a major problem in many countries, including China, India, the United States, Australia, and Russia (2). In India, the total estimated area affected by salinity is 6.73 million hectares, and the states of Gujarat, Uttar Pradesh, Maharashtra, West Bengal, and Rajasthan together account for approximately 75% of salt-affected soils in India (3). Soil salinity affects crop plants in all aspects of crop development, including germination, plant growth, flowering, fruiting, and seed setting (4). The role of microorganisms in ameliorating salt stress in various crops has been reported in various studies (5–7).

Chromohalobacter salexigens ANJ207 was isolated from salt crystals deposited in the pipelines of the Indian Council of Agricultural Research–National Bureau of Agriculturally Important Microorganisms (ICAR-NBAIM) in Kushmaur, Mau, India (25.8982°N and 83.4891°E). The strain was isolated using nutrient broth and various concentrations of NaCl (2 to 35% [wt/vol]) with overnight incubation at 37 ± 2°C. Based on differences in the colony morphology and Gram staining, an individual bacterial strain was selected for serial dilution. The isolate was grown in nutrient broth at 37°C in the presence of 30% NaCl, and total genomic DNA was extracted using the method described by Sharma et al. (8). A TruSeq Nano DNA library kit (Illumina) was used for library preparation (9). The genome was sequenced with an Illumina HiSeq 2000 platform, generating 30.4 million (30,401,402) paired-end reads (read length, 101 bp; insert size, 411 bp) totaling 3.07 Gb in size. The Next-Generation Sequencing Quality Control (NGS QC) Toolkit version 2.3 (10) was used to filter high-quality data (at a Phred score of ≥20), and 26.75 million high-quality reads were obtained. Primary assembly was done using Velvet v.1.2.10 (11), generating a total of 116 contigs with a k-mer length of 73. Initial assembly generated a 3,666,171-bp contig. Assembly quality was assessed with *N*_50_ contig size, and the *N*_50_ contig size was 134,580 bases. Scaffolding was then done using the program SSPACE v.3.0 (12), generating a total number of 33 scaffolds with a genome size of 3,664,372 bp (G+C content, 63.71%). Final assembly was done after removing scaffolds smaller than 142 bp using CONTIGuator v.2.7 (13), which generated a 3,666,372-bp genome in 13 scaffolds. Genome annotation was done using Rapid Annotation using Subsystems Technology (RAST) v.2.0 (14), which predicted a total number of 3,406 genes distributed over 8 scaffolds. Of the 3,406 predicted genes, 3,344 were protein-coding genes (2,868 characterized proteins and 506 hypothetical/putative proteins) and 62 were non-protein-coding genes. The total number of tRNA-coding genes was 54, and the total number of rRNA-coding genes was 3.

Overall, the genome analysis of Chromohalobacter salexigens ANJ207 will provide an elementary platform for future studies toward complete understanding of the functions of this halotolerant bacterium. Moreover, comparisons among the completely sequenced genomes of Chromohalobacter will facilitate rendering of new insights into evolutionary changes in *Chromohalobacter* spp. and reveal the genetic adaptations of these bacteria to survive under hypersaline conditions and the effectiveness of strains as plant growth promoters.

### Data availability.

This whole-genome shotgun project has been deposited in the NCBI repository under GenBank accession number MZZK00000000 and assembly accession number GCA_004102695 (BioProject identifier PRJNA377371). Short-read data have been submitted to the SRA under run accession number SRR8517641.
